# Why are social interactions found quickly in visual search tasks?

**DOI:** 10.1016/j.cognition.2020.104270

**Published:** 2020-07

**Authors:** Tim Vestner, Katie L.H. Gray, Richard Cook

**Affiliations:** a Department of Psychological Sciences, Birkbeck, University of London, London, UK; b School of Psychology and Clinical Language Sciences, University of Reading, Reading, UK; c Department of Psychology, University of York, York, UK

**Keywords:** Social interaction, Visual search, Direction cues, Social attention, Arrows

## Abstract

When asked to find a target dyad amongst non-interacting individuals, participants respond faster when the individuals in the target dyad are shown face-to-face (suggestive of a social interaction), than when they are presented back-to-back. Face-to-face dyads may be found faster because social interactions recruit specialized processing. However, human faces and bodies are salient directional cues that exert a strong influence on how observers distribute their attention. Here we report that a similar search advantage exists for ‘point-to-point’ and ‘point-to-face’ target arrangements constructed using arrows – a non-social directional cue. These findings indicate that the search advantage seen for face-to-face dyads is a product of the directional cues present within arrangements, not the fact that they are processed as social interactions, *per se*. One possibility is that, when arranged in the face-to-face or point-to-point configuration, pairs of directional cues (faces, bodies, arrows) create an attentional ‘hot-spot’ – a region of space in between the elements to which attention is directed by multiple cues. Due to the presence of this hot-spot, observers' attention may be drawn to the target location earlier in a serial visual search.

## 1 Introduction

Social perception research has traditionally sought to understand the cognitive and neural mechanisms that allow us to perceive faces (Duchaine & Yovel, 2015; Freiwald, Duchaine, & Yovel, 2016), facial expressions (Jack & Schyns, 2017), body shapes (Peelen & Downing, 2007; Ramsey, 2018), body postures (Reed, Stone, Bozova, & Tanaka, 2003), actions and kinematics (Blake & Shiffrar, 2007). While research in this tradition has made considerable progress elucidating the visual perception of *individuals*, we know relatively little about the visual perception of *social interactions*. Given the adaptive value of accurate interaction interpretation (e.g., for social learning and navigating our social environment), this paucity of knowledge is remarkable.

In the last few years, cognitive scientists have started to address this gap in our understanding. Early findings suggest that the observation of interacting individuals recruits regions of cortex, including superior temporal (e.g., Isik, Koldewyn, Beeler, & Kanwisher, 2017; Walbrin, Downing, & Koldewyn, 2018) and lateral occipital areas (Abassi & Papeo, 2020), that are not engaged by non-interacting individuals. Similarly, social interaction displays appear to recruit perceptual integration mechanisms that are not engaged by non-interacting individuals. For example, where two people appear to be interacting, the facial emotion of one individual alters the perceived expression of the other (Gray, Barber, Murphy, & Cook, 2017) and the individuals are remembered as standing closer together than they actually were (Vestner, Tipper, Hartley, Over, & Rueschemeyer, 2019). These perceptual and mnemonic biases are not seen for non-interacting individuals.

Recently, there has been considerable interest in reports that, when viewed from third-person perspectives, interacting individuals are found faster in visual search tasks than non-interacting individuals. Specifically, when asked to find a target dyad, amongst non-interacting ‘distractors’, participants respond faster when the individuals in the target dyad are shown face-to-face (suggestive of interaction), than when presented back-to-back (Papeo, Goupil, & Soto-Faraco, 2019; Vestner et al., 2019). This is the case when the face-to-face and back-to-back targets are closely matched for distractor-similarity. These findings are potentially important as they suggest that the visual processing of social interactions may be given greater priority, or achieved with greater efficiency, than the processing of non-interacting individuals.

In the present study, we sought a better understanding of the visual search advantage reported for social interactions. Face-to-face dyads may be found faster than back-to-back dyads because one is processed as a social interaction and the other is not (Papeo et al., 2019; Vestner et al., 2019). However, human faces and bodies are salient directional cues that exert a strong influence on how observers distribute their attention (Frischen, Bayliss, & Tipper, 2007; Nummenmaa & Calder, 2009). The different stimulus arrangements employed in these studies (face-to-face vs. back-to-back) not only affect the perception of social interaction, but they also alter the configuration of these directional cues. We sought to determine whether the search advantage for face-to-face dyads is a product of social interaction processing (i.e., domain-specific visual processing engaged selectively or preferentially by social interactions) or the configuration of directional cues contained therein.

## 2 Experiment 1

First, we sought to replicate the search advantage for face-to-face dyads, using a procedure similar to that described by Vestner et al. (2019). In Experiment 1, target and distractor dyads depicted individuals in profile such that their head, face and body were visible (Vestner et al., 2019). In Experiment 2, we sought to determine whether the search advantage could be replicated using profile views of actors' heads and faces only.

The experiments described were conducted online, an approach that is increasingly common. Carefully-designed online tests of cognitive and perceptual processing can yield high-quality data, indistinguishable from that collected in the lab (e.g., Crump, McDonnell, & Gureckis, 2013; Germine et al., 2012; Woods, Velasco, Levitan, Wan, & Spence, 2015).

### 2.1 Method and results

Fifty participants (31 female, 19 male) with an age range of 18 to 57 years (*M*_age_ = 28.0, *SD*_age_ = 9.8) were recruited through Prolific (https://www.prolific.com). Participants were only invited if they indicated that they lived in the United Kingdom, were aged between 18 and 65 years-old, and had a Prolific record of at least 75% satisfactorily completed experiments. Sample size (*N* = 50) was determined a priori, informed by a power analysis conducted assuming an effect size similar to that seen previously (Papeo et al., 2019; Vestner et al., 2019). Ethical clearance was granted by the local ethics committee and the experiment was conducted in line with the ethical guidelines laid down in the 6th (2008) Declaration of Helsinki. All participants gave informed consent.

Images of eight individuals (4 female, 4 male) viewed in profile were sourced from the Adobe Stock Service. Images were normed to a height of 350 pixels. For each image, a mirror-image was created so that a given individual could appear facing left or right. The experiment itself was coded using Unity3D (Version 2018.3.7f1), compiled to WebGL and hosted on an Amazon Lightsail server. This allowed the experiment to run in a participant's browser and response times (RTs) to be recorded locally without being influenced by variations in data transmission speed to the server. Piloting confirmed that this online procedure produces similar RT distributions to those seen in the lab.

The experiment consisted of two blocks of 50 trials (45 experimental trials plus 5 catch trials), completed in a counterbalanced order. Each trial started with a black cross that divided the white display into four quadrants. Participants initiated the onset of the search array by pressing and holding down spacebar. While spacebar was held down, a pair of individuals would appear in each quadrant. Each dyad was made up of two same-sex individuals chosen from the pool of stimuli by the experimental program. The same two individuals featured in all four dyads presented on a given trial.

**Fig. 1:**
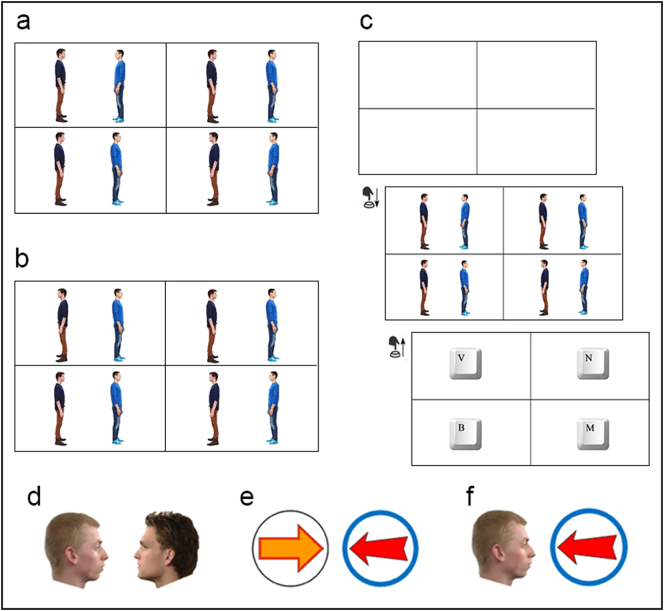
The search array employed in Experiment 1 on (a) facing trials and (b) non-facing trials. (c) The trial sequence from Experiment 1. (d–f) Examples of target dyads from Experiment 2–4, respectively.

Experimental trials presented a target dyad, and three distractor dyads. In one block, the target dyads were face-to-face (Fig. 1a); in the other, the target dyads were back-to-back (Fig. 1b). The three distractor dyads presented the same individuals both looking in the same direction, either left or right. Each trial featured at least one distractor pair facing left and one facing right. Participants were instructed to let go of spacebar as soon as they found the target dyad. As soon as they let go, the search array was replaced by a display prompting participants to identify the target location by making one of four keypress responses (Fig. 1c). Participants were therefore unable to continue their search after the release of the spacebar (Meegan & Tipper, 1998). RTs measure the interval from when spacebar was pressed to when it was released.

Catch trials did not present a target dyad. Instead, search arrays comprised only distractor dyads; two facing leftwards, two facing rightwards. On catch trials, participants were instructed to hold down spacebar until all the pairs disappeared (after 5 s). Catch trials were included to discourage participants from releasing spacebar before the target pair had been found on test trials.

**Fig. 2:**
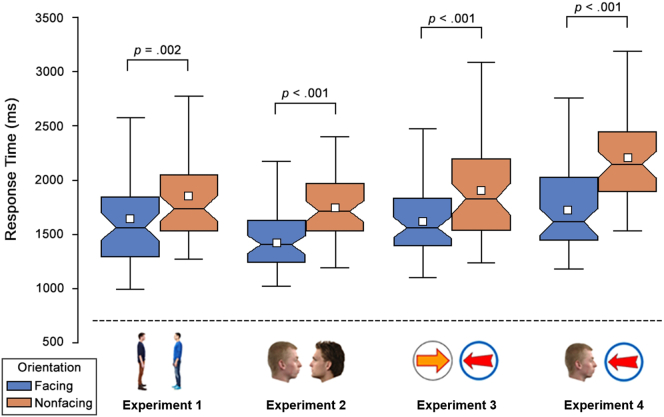
Response time distributions for Experiments 1–4. Boxes indicate inter-quartile range. Notches indicate confidence interval of the median. Whiskers indicate 1.5 ∗ interquartile range. White squares denote the mean.

The RT distributions for the facing and non-facing targets can be seen in Fig. 2. Those trials where participants responded incorrectly (2%), or where they took longer than 6 s to respond (1.8%), were excluded from the analysis. All participants completed at least 6 of the 10 catch trials correctly. No-one was replaced or excluded. Consistent with the search advantage described previously, face-to-face targets (*M* = 1.67 s, *SD* = 0.51 s) were found significantly faster than back-to-back targets (*M* = 1.85 s, *SD* = 0.44 s) [*t*(49) = 3.21, *p* = .002, *d* = 0.45, CI_95%_ = 0.07, 0.29]. Unsurprisingly, the distribution of raw RTs shows a degree of positive skewing. We note however, that the search advantage was also seen when an inverse log transformation was applied to attenuate positive skewing [*t*(49) = 3.85, *p* < .001, *d* = 0.55, CI_95%_ = 0.02, 0.07].

## 3 Experiment 2

A further 50 participants (20 female, 30 male) with an age range of 19 to 52 years (*M*_age_ = 29.5, *SD*_age_ = 9.7) were recruited through Prolific. With the exception of the stimuli used (faces viewed in profile), Experiment 2 was identical to Experiment 1. The facial images used to create the target and distractor dyads were downloaded from the Radboud Face Database (Langner et al., 2010). Individuals were shown in profile with a neutral expression and forward gaze (Fig. 1d). Each dyad was made up of two same-sex individuals chosen by the experimental program from a pool of stimuli (4 female, 4 male). The same two individuals featured in all four dyads presented on a given trial.

The RT distributions for the facing and non-facing targets can be seen in Fig. 2. Those trials where participants responded incorrectly (1.9%), or where they took longer than 6 s to respond (1.1%), were excluded from the analysis. All participants completed at least 6 of the 10 catch trials correctly. No-one was replaced or excluded. Once again, we replicated the search advantage: face-to-face targets (*M* = 1.47 s, *SD* = 0.31 s) were found significantly faster than back-to-back targets (*M* = 1.77 s, *SD* = 0.39 s) [*t*(49) = 6.44, *p* < .001, *d* = 0.91, CI_95%_ = 0.21, 0.41]. The search advantage was also seen when an inverse log transformation was applied to attenuate positive skewing [*t*(49) = 6.45, *p* < .001, *d* = 0.91, CI_95%_ = 0.06, 0.11].

## 4 Experiment 3

In our first two experiments we replicated the previously reported search advantage for face-to-face dyads (Papeo et al., 2019; Vestner et al., 2019). In Experiment 3, we examined whether pairs of arrows that point towards each other (‘point-to-point’) were found faster than target displays where the arrows point away from each other (‘base-to-base’). Like faces and bodies, arrows cue the attention of observers in a fast and automatic (i.e., hard to inhibit) manner (Kuhn & Kingstone, 2009; Tipples, 2002). If the search advantage for face-to-face dyads is a product of directional cueing by the constituent faces and bodies, a similar advantage should be seen for point-to-point arrangements constructed using arrows. If the effect is a product of specialized interaction processing, however, it should not be possible to replicate the search advantage with arrangements of non-social direction cues (e.g., Santiesteban, Catmur, Hopkins, Bird, & Heyes, 2014).

### 4.1 Methods and results

A further 50 participants (23 female, 26 male, 1 non-binary) with an age range of 19 to 54 years (*M*_age_ = 30.5, *SD*_age_ = 9.6) were recruited through Prolific. The experiment was identical to Experiments 1 and 2 except that the target and distractor arrangements each comprised two arrows. The same two arrows were employed throughout the procedure (Fig. 1e). We chose to use block arrows placed in circles to minimise the influence of other gestalt grouping principles, such as proximity or good closure (Coren & Girgus, 1980). We tried to make the arrows visually interesting to facilitate comparison with bodies (Experiment 1) and faces (Experiment 2).

The RT distributions for the point-to-point and base-to-base targets can be seen in Fig. 2. Those trials where participants responded incorrectly (2%), or where they took longer than 6 s to respond (1.5%), were excluded from the analysis. All participants completed at least 6 of the 10 catch trials correctly. No-one was replaced or excluded. We saw clear evidence of the same search advantage: point-to-point targets (*M* = 1.64 s, *SD* = 0.36 s) were found significantly faster than base-to-base targets (*M* = 1.89 s, *SD* = 0.45 s) [*t*(49) = 4.14, *p* < .001, *d* = 0.59, CI_95%_ = 0.13, 0.37]. This search advantage was also seen when an inverse log transformation was applied to attenuate positive skewing [*t*(49) = 4.42, *p* < .001, *d* = 0.63, CI_95%_ = 0.03, 0.09].

## 5 Experiment 4

The search advantage found for point-to-point targets (Experiment 3) closely resembles the search advantage seen for face-to-face dyads (Experiments 1 and 2). This suggests that the effect described is a product of the directional cues present within target arrangements, not specialized interaction processing. In its strongest form, the directional cueing account predicts that the search advantage should be found for any target pair comprising directional cues arranged to cue each other, irrespective of the nature or visual appearance of the individual elements. Alternatively, it is possible that the presence of two elements with similar characteristics (i.e., two faces or two arrows) affords greater symmetry and may encourage binding based on Gestalt cues (Coren & Girgus, 1980). It is possible that this inter-element symmetry is necessary to produce the search advantage for face-to-face and point-to-point arrangements.

We sought to distinguish these possibilities in Experiment 4. We employed mixed-element target displays consisting of a face and an arrow to determine whether ‘point-to-face’ targets are found faster than ‘back-to-base’ targets despite the different visual and semantic features of the constituent elements. If the visual search advantage is solely attributable to directional cueing, we should again find a significant RT advantage for point-to-face pairs over back-to-base pairs. If, however, some degree of elemental symmetry is required, no search advantage should be seen with these mixed element displays.

### 5.1 Methods and results

A further 50 participants (28 female, 22 male) with an age range of 19 to 60 years (*M*_age_ = 29.9, *SD*_age_ = 8.2) were recruited through Prolific. The experiment was identical to Experiments 1–3 except that target and distractor arrangements each comprised an arrow and a face (Fig. 1f).

The RT distributions for the point-to-face and base-to-back targets can be seen in Fig. 2. Those trials where participants responded incorrectly (1.8%), or where they took longer than 6 s to respond (1.3%), were excluded from the analysis. All participants completed at least 6 of the 10 catch trials correctly. No-one was replaced or excluded. Once again, we saw evidence of the search advantage: point-to-face targets were found significantly faster (*M* = 1.78 s, *SD* = 0.48 s) than base-to-back targets (*M* = 2.21 s, *SD* = 0.45 s) [*t*(49) = 5.84, *p* < .001, *d* = 0.83, CI_95%_ = 0.29, 0.58]. The search advantage was also seen when an inverse log transformation was applied to attenuate positive skewing [*t*(49) = 6.61, *p* < .001, *d* = 0.93, CI_95%_ = 0.07, 0.13].

## 6 Experiment 5

During peer-review it was put to us that the search advantage seen for arrows may be less robust than the advantage seen for arrangements constructed from human faces and bodies. If social interactions recruit domain-specific visual processing, the search advantage seen for face-to-face arrangements may be relatively insensitive to procedural differences such as the interleaving or blocking of trial type. In contrast, it was suggested that the search advantage seen for arrows may disappear if point-to-point and base-to-base trials were interleaved and not blocked (as in Experiments 3 and 4).

We sought to test this possibility in Experiment 5. One group of 50 participants (23 female, 26 male, 1 non-binary) with an age range of 18 to 60 years (*M*_age_ = 32.9, *SD*_age_ = 10.1) completed the search task with arrangements constructed from faces. A second group of 50 participants (15 female, 35 male) with an age range of 18 to 58 years (*M*_age_ = 29.7, *SD*_age_ = 9.5) completed the search task with arrangements constructed from arrows. In Experiments 1–4, trial type (face-to-face vs. back-to-back or point-to-point vs. base-to-base) was blocked. In Experiment 5, however, trial type was interleaved. In both tasks, participants were told that target pairs would comprise two elements pointing in different directions, while distractor pairs would comprise two elements pointing in the same direction. As in Experiment 2, dyads in the face task were constructed from a pool of 8 images (4 male, 4 female). To ensure comparability, dyads in the arrows task were also constructed from a pool of 8 exemplars.

**Fig. 3:**
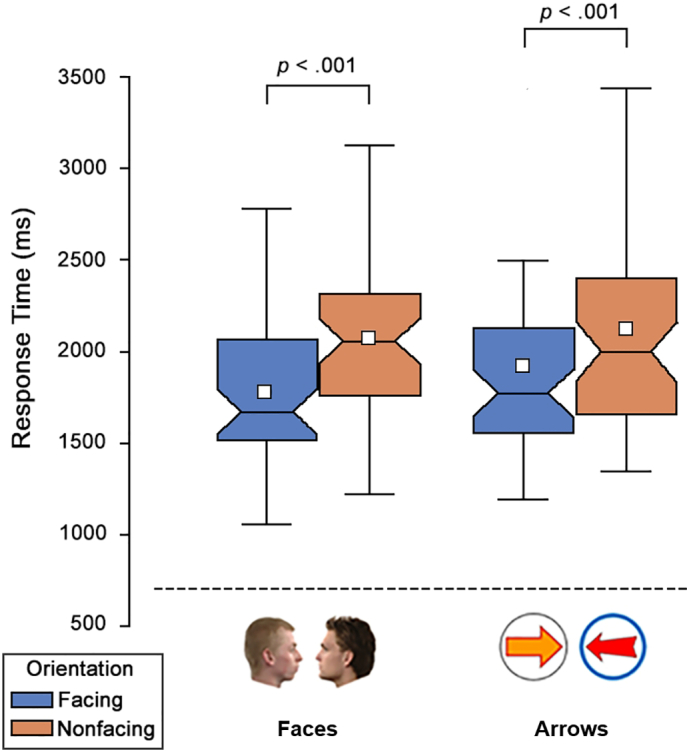
Response time distribution for Experiment 5. Boxes indicate inter-quartile range. Notches indicate confidence interval of the median. Whiskers indicate 1.5 ∗ interquartile range. White squares denote the mean.

The RT distributions seen for faces (face-to-face vs. back-to-back) and arrows (point-to-point vs. base-to-base) can be seen in Fig. 3. Those trials where participants responded incorrectly (faces: 2.5%; arrows: 1.7%), or where they took longer than 6 s to respond (faces: 1.2%; arrows: 1.9%), were excluded from the analysis. All participants completed at least 6 of the 10 catch trials correctly. One participant (faces task) was replaced prior to analysis having responded on all catch trials.

Despite the change of procedure, we replicated the search advantage for facing targets in both the face and arrow tasks. In the face task, face-to-face targets were found significantly faster (*M* = 1.79 s, *SD* = 0.48 s) than back-to-back targets (*M* = 2.10 s, *SD* = 0.50 s) [*t*(49) = 9.29, *p* < .001, *d* = 1.31, CI_95%_ = 0.24, 0.37]. In the arrow task, point-to-point targets were found significantly faster (*M* = 1.93 s, *SD* = 0.73 s) than base-to-base targets (*M* = 2.14 s, *SD* = 0.79 s) [*t*(49) = 6.99, *p* < .001, *d* = 0.99, CI_95%_ = 0.15, 0.27]. The search advantage for face-to-face targets [*t*(49) = 9.06, *p* < .001, *d* = 1.28, CI_95%_ = 0.05, 0.08] and base-to-base targets [*t*(49) = 7.48, *p* < .001, *d* = 1.07, CI_95%_ = 0.03, 0.06] seen in the face and arrow tasks, respectively, was also evident when an inverse log transformation was applied to attenuate positive skewing.

## 7 Discussion

Having successfully replicated the search advantage for face-to-face dyads (Experiments 1 and 2), we went on to find a similar search advantage for point-to-point target arrangements constructed using arrows (Experiment 3). Next, we replicated the effect with mixed-element displays comprising a face and an arrow, showing that the search advantage is also seen where the individual cues differ in their visual features and semantic content (Experiment 4). Finally, we confirmed that the search advantage for face-to-face and point-to-point targets is seen regardless of whether trial type is blocked or interleaved (Experiment 5). Together, these findings indicate that the search advantage found for face-to-face dyads is a product of the directional cues present within arrangements, not domain-specific social interaction processing.

The different configuration of direction cues in facing and non-facing arrangements may influence the way observers attend to displays in several ways. However, one possible explanation for the search advantage studied here is the creation of a hot-spot – a relatively small region of space to which attention is directed by multiple cues. The region in between the facing cues is the only portion of the search display that is cued by multiple elements (e.g., two faces), and may therefore be subject to additive or super-additive cuing effects. Due to the presence of this hot-spot, observers' attention may be drawn to the target location relatively early in a serial visual search. The opposite is true for the non-facing arrangements. In the back-to-back, base-to-base, or base-to-back arrangements, neither of the elements cue this central region. In fact, the individual elements direct observers' attention away from the target location. As a result, observers may find the target location later in a serial visual search.

We have argued that facing dyads are found faster in visual search tasks than non-facing dyads because of the differential arrangements of directional cues contained within target displays. However, it is not our intention to imply that the search effect is seen with any stimulus with a front-back axis. Vestner et al. (2019) failed to observe the effect with wardrobes and upside-down faces, despite the fact that these stimuli both have a canonical ‘front’ and ‘back’. It appears that the effect is seen only with very strong directional cues where the orienting effect is hard to inhibit (Kuhn & Kingstone, 2009; Tipples, 2002). In this context, it is important to note that inverted faces produce weaker gaze cueing effects compared to upright faces; in other words, upside-down faces are less effective directional cues, than upright faces (e.g., Langton & Bruce, 1999). That the search advantage is absent for wardrobes and inverted faces (Vestner et al., 2019) also argues against an account based on low-level features, including symmetry (e.g., Wolfe & Friedman-Hill, 1992).

While the study of social interaction perception is an exciting new field, paradigms are still being refined. A multitude of published studies have already employed the face-to-face vs. back-to-back manipulation to isolate the neural (Abassi & Papeo, 2020; Isik et al., 2017; Quadflieg, Gentile, & Rossion, 2015; Walbrin et al., 2018), perceptual (Gray et al., 2017; Papeo & Abassi, 2019; Papeo et al., 2019; Papeo, Stein, & Soto-Faraco, 2017; Strachan, Sebanz, & Knoblich, 2019), and mnemonic (Vestner et al., 2019) processes recruited by interacting individuals. Our results indicate that the visual search advantage found for face-to-face, relative to back-to-back dyads, is not attributable to domain-specific social interaction processing; rather, it appears to be a product of the different arrangement of directional cues contained within facing and non-facing targets. In future studies, authors may wish to examine the contribution of attentional cueing and engagement to putative neural (e.g., Abassi & Papeo, 2020), perceptual (e.g., Gray et al., 2017; Papeo et al., 2017), and mnemonic (e.g., Vestner et al., 2019) markers of interaction processing that manifest disproportionately when observers view face-to-face, relative to back-to-back arrangements.

## CRediT authorship contribution statement

**Tim Vestner:** Conceptualization, Methodology, Software, Investigation, Formal analysis, Writing - original draft. **Katie L.H. Gray:** Conceptualization, Writing - review & editing. **Richard Cook:** Conceptualization, Resources, Writing - review & editing, Supervision.

## Appendix A Supplementary data

Supplementary material Image 1
